# Evaluation of the podoplanin/C-type lectin-like receptor-2 (CLEC-2) pathway as a mediator of platelet and coagulation activation in sickle cell disease

**DOI:** 10.1016/j.rpth.2025.103168

**Published:** 2025-09-01

**Authors:** Ivanio Teixeira Borba-Junior, Mayck da Silva Barbosa, Carla Roberta Peachazepi Moraes, Letícia Queiroz Silva, Irene Santos, Bruno Benites, Joyce M. Annichino-Bizzacchi, Fernando Ferreira Costa, Erich Vinicius De Paula

**Affiliations:** 1 Faculty of Medical Sciences, State University of Campinas, Campinas, Brazil; 2 Hematology and Hemotherapy Center, State University of Campinas, Campinas, Brazil

**Keywords:** CLEC-2, leukocyte–platelet aggregate, podoplanin, sickle cell disease, thromboinflammation

## Abstract

**Background:**

Sickle cell disease (SCD) is a condition characterized by a prothrombotic state attributed to the simultaneous activation of hemostasis and innate immunity, referred to as thromboinflammation. Previous studies have demonstrated that the podoplanin (PDPN)/C-type lectin-like receptor-2 (CLEC-2) pathway is an emerging and important element of the pathogenesis of conditions in which inflammation and thrombosis coexist, but no data is available regarding its role in SCD.

**Objectives:**

To explore the PDPN/CLEC-2 pathway in SCD and correlate it with parameters of disease severity.

**Methods:**

Fifty SCD patients (35 with SS genotype; 15 with SC genotype) and 25 healthy individuals were recruited. PDPN and CLEC-2 were assessed for both soluble and surface expression on cells and cell aggregates, along with other classical parameters of hemostasis and platelet activation. An *in vitro* study was performed to analyze the effect of anti-PDPN antibody on the formation of monocyte–platelet aggregates.

**Results:**

Circulating levels and expression of PDPN and CLEC-2 were higher in patients with SCD, particularly in those with genotype SS. The number of CD41^+^CLEC^+^ monocytes correlated with hemoglobin, D-dimer, von Willebrand factor, and PDPN^+^ monocytes. *In vitro*, PDPN blockade reduced both monocyte–platelet aggregate formation and platelet activation. Finally, patients with a history of vaso-occlusive crises presented a trend toward increased PDPN expression in monocytes (*P* = .06).

**Conclusion:**

Our findings suggest that the PDPN/CLEC-2 pathway may play an important role in the pathogenesis of thromboinflammation in SCD, especially in patients with the SS genotype.

## Essentials

• Thromboinflammation contributes to the hypercoagulable state in SCD.
• We evaluated the PDPN/CLEC-2 pathway in patients with SCD and healthy individuals.
• SS patients showed increased soluble and cellular PDPN/CLEC-2 expression.
• PDPN/CLEC-2 pathway may be part to the thromboinflammatory cascade in SCD.

## Graphical abstract

**Figure:**
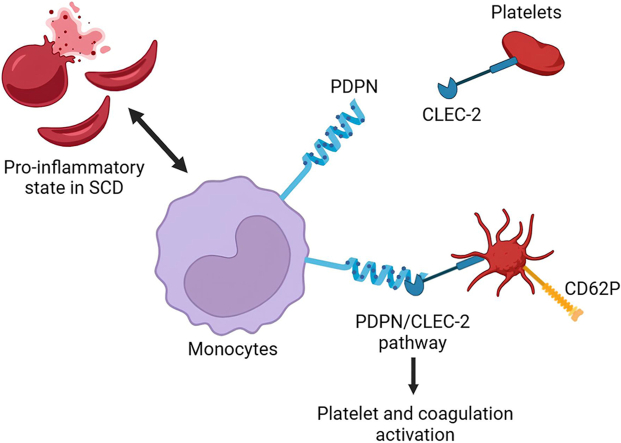

## 1 Introduction

Hypercoagulability is an important feature of sickle cell disease (SCD) [1], and its pathophysiology is related to the concomitant activation of hemostasis and innate immunity in a process known as thromboinflammation [2]. This concept fits the SCD model as hemostasis activation is an integral part of this inflammatory response, stimulated by specific conditions such as the release of free heme or free hemoglobin S and ischemia-reperfusion injury, among others [3,4]. The prothrombotic state of SCD is evidenced by elevated levels of hemostasis activation biomarkers, such as D-dimer, thrombin-antithrombin, and von Willebrand factor (VWF) [1,5], as well as by a higher incidence of venous thromboembolism [6,7]. Moreover, several potential pathways of thromboinflammation have already been described in SCD such as NETosis [8] and intravascular tissue factor expression [9], among others [10,11]. Despite this evidence, therapeutic targets capable of effectively downregulating this prothrombotic state in SCD are yet to be determined.

Podoplanin (PDPN) is a protein primarily expressed in lymphatic vessels but is also found in hematopoietic cells such as monocytes [12]. The only known ligand for PDPN is CLEC-2 (C-type lectin-like receptor-2), which is primarily expressed on platelets [13]. Evidence suggests that the PDPN/CLEC-2 pathway is associated with thrombosis [[14], [15], [16]]. Here, we explored the expression of PDPN and CLEC-2 in plasma and cells that participate in thromboinflammation in SCD patients.

## 2 Methods

### 2.1 Study population and clinical data

This was a prospective cohort study conducted with patients from the hemoglobinopathy outpatient clinic at Hemocentro Unicamp, as well as healthy controls. The inclusion criteria were age >18 years, confirmed diagnosis of SCD, and disease in a steady state, as defined as the absence of crises or transfusions in the last 30 days. Exclusion criteria included the presence of signs or symptoms of inflammatory disease in the last 10 days, use of anticoagulants, and a diagnosis of cancer.

Patients were recruited from May 2022 to February 2023, and all patients who met the inclusion criteria were invited to participate until the predetermined target of 50 patients was reached (35 with SS genotype; 15 with SC genotype). Additionally, 25 healthy, asymptomatic individuals were recruited during the same period from the same geographic region. The study was conducted in accordance with the Declaration of Helsinki and approved by the institutional ethics committee (CAAE: 53121421.0.0000.5404). Clinical and laboratory data were collected from the hospital’s electronic medical records. The number of vaso-occlusive crises (VOCs) in the past year was determined based on emergency room visits in which the patient remained for ≥4 hours.

### 2.2 Sample collection and processing

Samples were collected in ethylenediaminetetraacetic acid (EDTA) tubes (BD Vacutainer) and 3.2% sodium citrate tubes (BD Vacutainer) immediately after inclusion in the study. Samples were processed within 2 hours, and plasma was obtained after centrifugation at 2500*g* for 15 minutes and then frozen at −80 °C until analysis. To obtain platelet-free plasma, the citrate tubes underwent double centrifugation.

### 2.3 Laboratory evaluation of hemostasis

VWF antigen and VWF activity were measured in an automated coagulometer (ACL TOP 550 CTS; Instrumentation Laboratory) using commercial assays available from the same manufacturer (HemosIL reagents). D-dimer was measured using an immunoturbidimetric assay (Innovance D-Dimer; Siemens Healthcare).

### 2.4 Determination of soluble PDPN and CLEC-2 levels

PDPN and CLEC-2 levels were measured in EDTA plasma using commercial ELISA kits (RayBiotech cat. ELH-PDPN and ELH-CLEC, respectively). Optical density was measured at 450 nm in a plate reader (Multiskan GO; Thermo Fisher Scientific).

### 2.5 Expression of PDPN and CLEC-2 on circulating cells

For flow cytometry, 100 μL of EDTA whole blood was used immediately after collection from patients and healthy controls. Two tubes were prepared for each patient with the following antibodies. The first tube included anti-CD45 (PerCP, BioLegend cat. 982318), anti-CD62P (FITC, BioLegend cat. 986404), anti-PDPN (PE, BioLegend cat. 337004), and anti-CLEC-2 (APC, BioLegend cat. 372006); the second tube included anti-CD45 (PerCP, BioLegend cat. 982318), anti-CD41 (FITC, BD Biosciences cat. 555466), and anti-CLEC-2 (APC, BioLegend cat. 372006). After incubation for 30 minutes at room temperature, protected from light, red blood cells were lysed using a lysing and fixing solution for 15 minutes (BioLegend cat. 422401). Samples were centrifuged (540*g*, 5 minutes), the supernatant was discarded, and the cells were then washed with 2 mL of solution (phosphate-buffered saline with 0.01% bovine serum albumin). They were centrifuged again, the supernatant was discarded, and the cells were resuspended in 300 μL of solution, with 100,000 events acquired on a flow cytometer (BD FACSCalibur). Additionally, the Amnis flow cytometer (Luminex) was used for image cytometry.

Cell populations were separated using forward scatter (size) and side scatter (granularity) from CD45^+^ cells (Supplementary Figure 1). Monocytic and granulocytic cell gates were analyzed, and the results are described as percentages. For internal control, compensation was performed with separate antibodies, fluorescence minus one, and an isotype control. Data were analyzed using FlowJo software (BD Biosciences).

### 2.6 Evaluation of monocyte–platelet aggregate formation via PDPN/CLEC-2

Peripheral blood mononuclear cells were isolated from the blood of healthy individuals using a density gradient with Ficoll (GE Healthcare cat. 17144002). For the selective isolation of monocytes, cells were incubated for 2 hours in Dulbecco’s modified Eagle’s medium (Lonza cat. 12604Q) at 37 °C and 5% CO_2_, allowing monocytes to adhere to the bottom of the plate. Subsequently, the supernatant containing non-adherent cells was carefully removed, and fresh Dulbecco’s modified Eagle’s medium with 10% fetal bovine serum (Sigma) was added. PDPN expression was observed after 3 days of culture at 37 °C and 5% CO_2_, during which monocytes showed an average PDPN expression of 30% (Supplementary Figure 2).

In a paired study, monocytes were blocked with antibodies (IgG - control group) and anti-PDPN (1:1000) (Biolegend cat. 337002) for 30 minutes. Afterward, the cells were washed and centrifuged, and monocytes were resuspended in phosphate-buffered saline. Monocytes (5 × 10^5^) were then incubated with 300 μL of platelet-rich plasma for 10 minutes at 37 °C in a water bath. Subsequently, the samples were stained with antibodies (anti-CD14, -CD41, and -CD62P; Biolegend) for 20 minutes at room temperature, then fixed, washed, and analyzed by flow cytometry (BD FACSCalibur). Data were analyzed using FlowJo software (BD Biosciences).

### 2.7 Statistical analysis

Quantitative data are expressed as medians, percentiles, and mean ± SD. Comparisons were made using the Mann–Whitney U-test, Wilcoxon test, or Student’s *t*-test, according to the distribution and nature of the variables. Correlations were evaluated by Spearman’s tests. *P* < .05 was considered statistically significant. All statistical analyses were performed using SPSS version 26 (IBM) or GraphPad Prism 8.0 Software (GraphPad Inc).

## 3 Results

**Figure 1:**
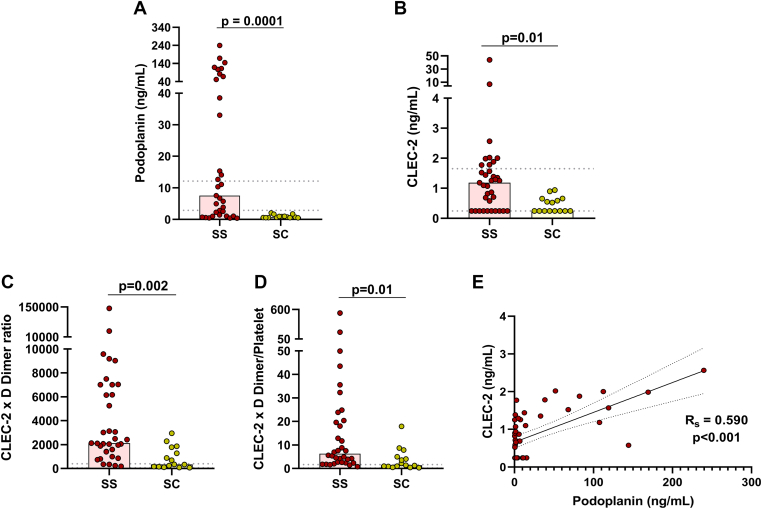
Assessment of plasma levels of podoplanin and C-type lectin-like receptor-2 (CLEC-2). (A) Soluble podoplanin levels are higher in sickle cell disease genotype SS patients than in genotype SC patients. (B) Soluble CLEC-2 levels are higher in SS patients than in SC patients. (C) CLEC-2 multiplied by D-dimer is increased in SS patients compared with SC patients. (D) CLEC-2 multiplied by D-dimer divided by platelet count is also increased in SS patients compared with SC patients. (E) Positive correlation between soluble CLEC-2 and soluble podoplanin (Mann–Whitney U-test; dotted lines represent the 95% CI of healthy individuals; Spearman correlation coefficients were calculated according to distribution of data from SCD patients).

The clinical and laboratory characteristics of the study population are described in the Table. Circulating levels of PDPN and CLEC-2 were higher in homozygous patients (SCD-SS) than in heterozygous patients (SCD-SC) (Figure 1A, B). We also explored the ratios of CLEC-2 levels to platelet count and D-dimer, which have been used as biomarkers of microvascular thrombosis [17,18], and found they were increased in SCD-SS patients (Figure 1C, D). In addition, we observed a positive correlation between soluble PDPN (sPDPN) and soluble CLEC-2 (sCLEC-2) (Figure 1E).

**Table:** Clinical and laboratory parameters of the study population.

Parameter	SS patients (*n* = 35)	SC patients (*n* = 15)	Healthy individuals (*n* = 25)	*P*
Age, y	45.1 ± 10.4	50.4 ± 12.9	39.7 ± 11.4	.29
Male:female	17:18	6:9	11:14	.75
Using hydroxyurea	30 (85.7%)	2 (14.2%)	-	.0001
VOC/ACS in last year	7 (20%)	0	-	.0001
Retinopathy	9 (25.7%)	9 (60%)	-	.02
HbF, %	16.6 (8.8-21.7)	0.55 (0.42-1.52)	-	.0001
Hb, g/dL	8.96 ± 1.76	12.0 ± 1.38	14.7 ± 1.34	.0001
Leucocytes, 10^9^/L	6.16 ± 1.7	7.95 ± 2.4	6.45 ± 1.84	.009
Neutrophils, 10^9^/L	3.25 ± 1.25	4.98 ± 2.18	3.89 ± 1.47	.001
Monocyte, 10^9^/L	0.29 ± 0.15	0.39 ± 0.16	0.36 ± 0.12	.08
Reticulocytes, 10^9^/L	271.3 ± 121.4	212 ± 70.8	85.7 ± 32.4	.10
Platelets, 10^9^/L	385.7 ± 197.3	275.4 ± 154.8	247.6 ± 52.6	.06
LDH, U/L	313 (271.5-417.5)	178.5 (156.5-238.3)	-	.001
TB, mg/dL	1.72 (1.19-2.75)	1.05 (0.89-1.90)	-	.03
IB, mg/dL	1.42 (1.11-2.29)	1.27 (0.84-1.64)	-	.20
VWF:Ag, %	226.7 (194.4-238.9)	149.7 (132.3-238.2)	112.8 (98.5-136.4)	.0001
VWF:Act, %	164.2 ± 53.1	137.3 ± 67.1	97.3 ± 20.2	.0001
D-dimer, ng/mL	2643 (1461-5439)	1050 (640-2541)	182 (114.3-279.5)	.06

Values presented as n (%), mean ± SD, or median (IQR). All *P* values refer to the comparison between SS and SC patients.

ACS, acute chest syndrome; Hb, hemoglobin; HbF, fetal hemoglobin; IB, indirect bilirubin; LDH, lactate dehydrogenase; TB, total bilirubin; VOC, vaso-occlusive crisis; VWF:Act, von Willebrand factor activity; VWF:Ag, von Willebrand factor antigen.

**Figure 2:**
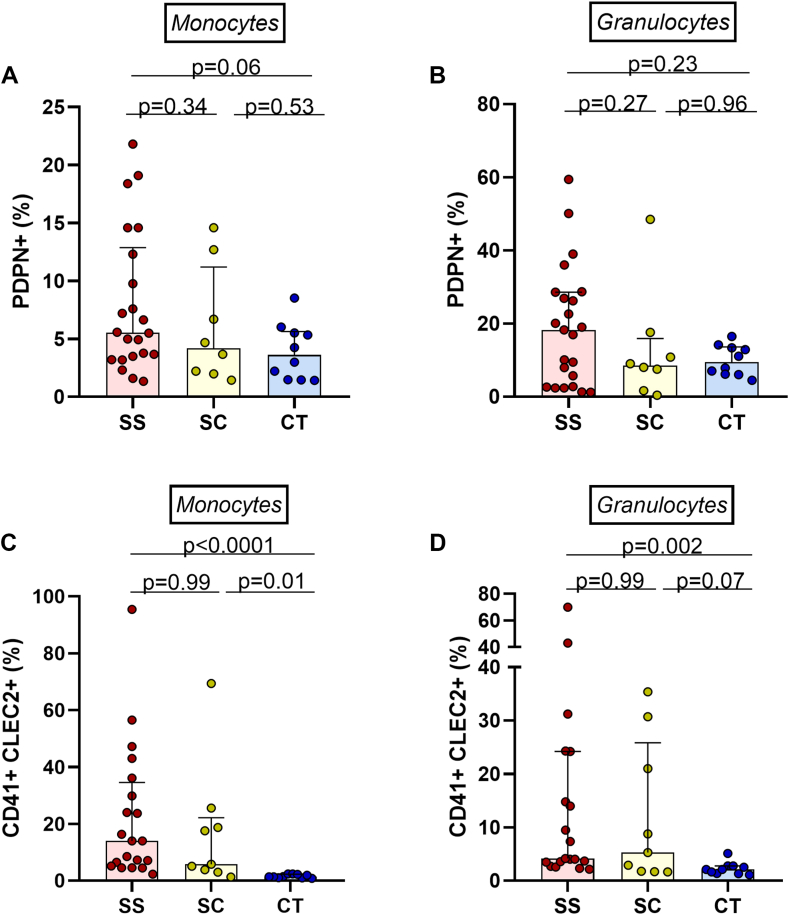
Evaluation of PDPN and CLEC-2 expression. (A, B) Assessment of PDPN expression in monocytic and granulocytic cells. (C, D) Increase in leukocyte–platelet aggregates (with monocytes and granulocytes) expressing CLEC-2 in patients with sickle cell disease (post-hoc Kruskal–Wallis test). CLEC-2, C-type lectin-like receptor-2; CT, control; SC, genotype SC; SS, genotype SS; PDPN, podoplanin.

These results were consistent with flow cytometry experiments in which a trend toward a higher PDPN expression was observed in monocytes from SCD-SS patients compared with those of healthy individuals (*P* = .06), but not in granulocytic cells (Figure 2A, B).

**Figure 3:**
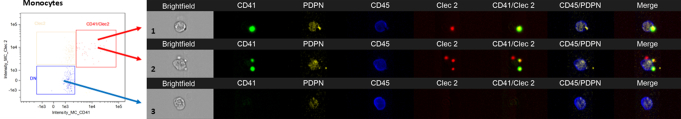
Assessment of PDPN and CLEC-2 expression in MPAs, the image shows that CLEC-2 is exclusively expressed on platelets; furthermore, PDPN is more evident on MPAs (panels 1 and 2 show MPAs, whereas panel 3 shows monocytes only). CLEC-2, C-type lectin-like receptor-2; DN, double negative; MPA, monocyte–platelet aggregate; PDPN, podoplanin.

The cellular expression of CLEC-2 was explored in leukocyte–platelet aggregates (LPAs) and was increased in SCD-SS patients compared to healthy individuals, both in monocyte–platelet aggregates (MPAs) and granulocyte–platelet aggregates (Figure 2C, D). Using imaging flow cytometry, we confirmed that the expression of CLEC-2 (red) was restricted to platelets, and that PDPN (yellow) was more evident in MPAs (Figure 3).

Of note, CLEC-2 expression in LPAs was associated with platelet activation measured by P-selectin (CD62P) expression, as evidenced by consistent positive correlations between CD41^+^CLEC-2^+^ monocytes and CD62^+^CLEC-2^+^ monocytes (*R*_s_ = 0.759; *P* < .001) and between CD41^+^CLEC-2^+^ granulocytes and CD62^+^CLEC-2^+^ granulocytes (*R*_S_ = 0.515; *P* = .001) (Supplementary Table 1).

**Figure 4:**
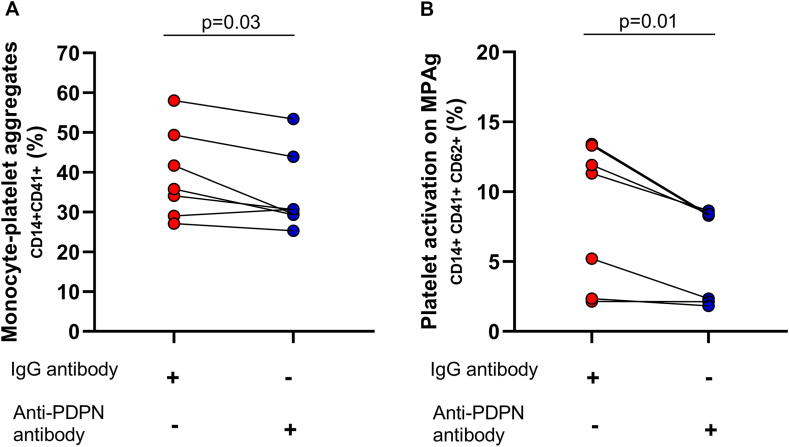
Role of PDPN blockade in the formation of monocyte–platelet aggregates (MPAs) and platelet activation mediated by the PDPN/CLEC-2 pathway. (A) Decreased MPA formation after PDPN blockade in monocytes using an anti-PDPN antibody. (B) Reduced platelet activation, assessed by CD62P expression in MPAs, after anti-PDPN treatment (Wilcoxon test). CLEC-2, C-type lectin-like receptor-2; PDPN, podoplanin.

To explore the role of PDPN in the formation and activation of these LPAs, we demonstrated that antibody-mediated PDPN blockade in cultured monocytes reduced the formation of LPAs, as well as platelet activation in these aggregates, as evidenced by CD62P expression (Figure 4A, B).

Finally, based on the results obtained for both soluble and cellular expression of PDPN and CLEC-2, we explored the association of these proteins with clinical and laboratory parameters of SCD severity. The frequency of CD41^+^CLEC-2^+^ monocytes was significantly correlated with hemoglobin (*R*_S_ = −0.507), reticulocyte count (*R*_S_ = 0.445), D-dimer (*R*_S_ = 0.643), VWF antigen (*R*_S_ = 0.495), CD62P^+^CLEC-2^+^ monocytes (*R*_S_ = 0.760), and PDPN^+^ monocytes (*R*_S_ = 0.494). In addition, PDPN^+^ monocytes were correlated with PDPN^+^ granulocytes (*R*_S_ = 0.664), CD62P^+^CD41^+^ monocytes (*R*_S_ = 0.683), and reticulocytes (*R*_S_ = 0.434) (Supplementary Table 1). Finally, a trend toward higher expression of PDPN in monocytes, granulocytes, and CLEC-2^+^ MPAs was observed in patients with a history of VOC (all *P* = .06; Supplementary Table 2).

## 4 Discussion

Several pathways involved in thromboinflammation have been associated with the pathogenesis of SCD [1]. However, no study has yet explored whether the PDPN/CLEC-2 pathway is involved in the pathogenesis of the inflammatory response in SCD. The main contribution of our study was to provide evidence of the association of the PDPN/CLEC-2 pathway with the pathogenesis of SCD by demonstrating its increased expression, both in serum and on circulating cells, as well as its association with markers of disease severity.

In recent years, several studies highlighted the importance of the PDPN/CLEC-2 pathway in platelet activation and thrombosis. Studies in animal models have demonstrated that thrombus formation is dependent on PDPN/CLEC-2 in both infection [16] and cancer [14]. Moreover, this pathway has also been associated with hypercoagulable states in humans, such as in patients with glioblastoma, in whom high tumor expression of PDPN was associated with an up to 5.7 times greater risk of venous thromboembolism [15]. Similar findings associating PDPN expression with coagulation and/or platelet activation have been reported in breast cancer [19] and acute promyelocytic leukemia [20].

In our study, we observed that circulating levels of sPDPN and sCLEC-2 in plasma are significantly elevated in homozygous SCD patients compared to those with hemoglobin SC disease. Elevated sPDPN levels have been previously reported in various types of cancer, where they were associated with thrombotic risk [19,21]. Similar results have also been reported in other inflammatory/prothrombotic conditions such as nephrotic syndrome [22] and COVID-19, although studies on COVID-19 have shown controversial results [23,24]. In regard to sCLEC-2, elevated levels have been reported in coronary artery disease [25], COVID-19 [26], and cerebral cancer [27]. Moreover, studies demonstrated that the combination of sCLEC-2 with platelet count and D-dimer increases the sensitivity and specificity for the diagnosis of microvascular thrombosis [17,18]. In our study, both the CLEC-2 × D-dimer ratio and the CLEC-2 × D-dimer/platelet count ratio were elevated in SS patients. Although these studies point to an association of elements of the PDPN/CLEC-2 pathway with thromboinflammatory conditions, the mechanisms of sPDPN and sCLEC-2 release are yet to be determined and could involve protease-mediated ectodomain shedding [28] or the release of extracellular vesicles, a mechanism that has been described for both CLEC-2 [29] and PDPN, especially in patients with cancer [30]. The relative contribution of the soluble or membrane-bound forms of these proteins to the thromboinflammatory cascade has not yet been described.

We also explored PDPN and CLEC-2 expression in circulating cells involved in the pathogenesis of SCD, as well as in LPAs, which have been shown to drive coagulation activation in inflammatory conditions [31] and to associate with disease severity in SCD [32,33]. The demonstration of the presence of PDPN in LPAs in patients with SCD, coupled with a trend toward a higher expression of PDPN (*P* = .06) in monocytes of SS patients than in those of healthy individuals reinforces the hypothesis that PDPN is an additional element of the thromboinflammatory response in SCD. Analogously, CLEC-2 expression was also present in LPAs containing both monocytic and granulocytic cells from patients with SCD, compared with those from healthy individuals. Moreover, in experiments with cultured monocytes, we showed that blocking PDPN with an anti-PDPN antibody reduced LPA formation and platelet activation. Together, these data represent an initial step toward understanding the mechanisms by which the PDPN/CLEC-2 pathway participates in the pathogenesis of SCD.

Finally, expression of both PDPN and CLEC-2 in LPAs was positively correlated with established laboratory markers of coagulation and platelet and endothelial activation, such as D-dimer, P-selectin, VWF, and reticulocyte counts, and showed a trend toward association with a history with VOC. Considering the established role of PDPDN/CLEC-2 in the pathogenesis of thrombosis in other contexts [15,16,20], our data support the concept that PDPN/CLEC-2 are involved in the thromboinflammatory response that underlies the prothrombotic state observed in SCD.

As mentioned above, higher levels of LPAs have been previously reported in SCD and are associated with platelet activation and VOC [32,33]. Recently, a mechanism by which LPA formation mediates coagulation activation involving tissue factor was further detailed in COVID-19 [34]. Thus, our findings pave the way for studies exploring whether the PDPN and CLEC-2 in LPAs represent novel therapeutic targets as well as biomarkers of disease severity in larger and independent cohorts.

Our study has limitations that need to be acknowledged, such as the relatively limited sample size. However, the inclusion criteria were well defined, and the collection and processing were well standardized. Another limitation is that the variable race/ethnicity, which is known to influence several immunological and metabolic traits, was not recorded, which should be accounted for when interpreting our results. Finally, our work did not explore the mechanisms that led to the release of soluble forms of PDPN and CLEC-2 or the activation of downstream elements of the PDPN/CLEC-2 pathway, which could have provided additional insights into the molecular mechanisms underlying our observations. Since this is the first study, to our knowledge, to explore the participation of this pathway in the pathogenesis of SCD, these experiments were beyond the scope of our study.

In conclusion, our results demonstrate that elements of the PDPN/CLEC-2 pathway are present in both the humoral and cellular compartments of the thromboinflammatory response of SCD patients and that levels of these elements segregate with laboratory parameters of disease severity. Together, our results present the first evidence that elements of the PDPN/CLEC-2 pathway are involved in the pathogenesis of SCD.

## Supplementary material

Supplementary Figures

Supplementary Tabels
