# Supplementary material for: Evaluation of the podoplanin/C-type lectin-like receptor-2 (CLEC-2) pathway as a mediator of platelet and coagulation activation in sickle cell disease
Source: Res Pract Thromb Haemost. 2025 Sep 1;9(6):103168. doi: 10.1016/j.rpth.2025.103168 (PMC12538053; doi:10.1016/j.rpth.2025.103168)
Supplement: Supplementary Tabels [file mmc2.docx]

**Supplementary table 1**. Correlation with laboratorial parameters.

| **Variable** | **Correlated with** | **Correlation coefficient** | **P** |
| --- | --- | --- | --- |
| **CLEC-2+ MPAs** | GPAs CLEC-2+  Monocytes PDPN+  CLEC2+CD62+ Monocytes  Hemoglobin  WBC  Platelets  Reticulocytes  D dimer  VWF:Ag  VWF Activity  sPDPN  sCLEC-2 | **0.850**  **0.494**  **0.760**  **-0.507**  -0.096  -0.010  **0.445**  **0.643**  **0.495**  **0.412**  -0.204  -0.038 | **0.0001**  **0.002**  **0.0001**  **0.001**  0.56  0.95  **0.006**  **0.0001**  **0.001**  **0.01**  0.22  0.81 |
| **CLEC-2+ GPAs** | Granulocytes PDPN+  CLEC2+CD62+ Granulocytes  Hemoglobin  WBC  Platelets  Reticulocytes  D dimer  VWF:Ag  VWF Activity  sPDPN  sCLEC-2 | **0.467**  **0.515**  -0.303  -0.177  -0.078  **0.371**  **0.541**  **0.415**  0.305  -0.161  -0.110 | **0.003**  **0.001**  0.06  0.29  0.64  **0.02**  **0.0001**  **0.01**  0.07  0.34  0.51 |
| **PDPN+ Monocytes** | Granulocytes PDPN+  CLEC2+CD62+ monocytes  Hemoglobin  WBC  Platelets  Reticulocytes  D dimer  VWF:Ag  VWF Activity  sPDPN  sCLEC-2 | **0.664**  **0.683**  -0.125  0.045  0.196  **0.434**  0.230  0.213  0.311  0.162  0.039 | **0.0001**  **0.0001**  0.44  0.78  0.23  **0.007**  0.15  0.18  0.056  0.32  0.81 |
| **PDPN+ Granulocytes** | CLEC2+CD62+ Granulocytes  Hemoglobin  WBC  Platelets  Reticulocytes  D dimer  VWF:Ag  VWF Activity  sPDPN  sCLEC-2 | **0.573**  0.074  0.155  0.131  0.218  0.136  0.159  0.190  -0.018  -0.049 | **0.0001**  0.65  0.33  0.42  0.18  0.39  0.32  0.24  0.91  0.76 |
| **sPDPN** | Hemoglobin  Fetal Hemoglobin  WBC  Platelets  Reticulocytes  D dimer  VWF:Ag  VWF Activity  sCLEC-2 | 0.129  **0.519**  **-0.284**  -0.040  -0.118  -0.140  -0.068  0.040  **0.598** | 0.27  **0.001**  **0.01**  0.73  0.33  0.24  0.57  0.74  **0.001** |
| **sCLEC-2** | Hemoglobin  Fetal Hemoglobin  WBC  Platelets  Reticulocytes  D dimer  VWF:Ag  VWF Activity | -0.073  0.293  -0.163  0.049  0.037  0.043  0.097  0.170 | 0.53  0.07  0.15  0.68  0.76  0.71  0.41  0.15 |

WBC, White blood cell; VWF:Ag, von Willebrand Factor: antigen; MPAs, Monocyte-platelet aggregates; GPAs, Granulocyte-platelet aggregates; sPDPN, soluble podoplanin; sCLEC-2, soluble CLEC-2; Correlation coefficients (Spearman, according to data distribution) were calculated using data from all participants (patients and healthy individuals).

**Supplementary Table 2**. Assessment of the PDPN/CLEC-2 pathway among patients with and without vaso-occlusive crisis in the past year.

|  | VOC | | P |
| --- | --- | --- | --- |
|  | ***Yes*** | ***No*** |  |
| sPDPN  (ng/mL) | 2.98  (0.86 - 25.3) | 2.0  (0.68 - 19.70) | 0.98 |
| sCLEC-2  (ng/mL) | 0.450  (0.245 - 0.996) | 0.867  (0.245 - 1.438) | 0.27 |
| PDPN+  Monocytes (%) | 12.3  (5.68 - 16.8) | 4.93  (2.75 - 8.49) | 0.06 |
| PDPN+  Granulocytes (%) | 39.0  (10.8 - 49.3) | 9.79  (2.58 - 23.5) | 0.06 |
| CLEC-2+  MPAs (%) | 25.6  (10.4 - 62.2) | 7.85  (4.69 - 23.69) | 0.21 |
| CLEC-2+  GPAs (%) | 35.4  (5.0 - 56.5) | 4.0  (2.55 - 14.8) | 0.06 |

VOC: vaso-occlusive crisis; MPAs, Monocyte-platelet aggregates; GPAs, Granulocyte-platelet aggregates; sPDPN, soluble podoplanin; sCLEC-2, soluble CLEC-2; Mann-Whitney test according to data distribution. Median (IQR).
