# Supplementary material for: Evaluation of the podoplanin/C-type lectin-like receptor-2 (CLEC-2) pathway as a mediator of platelet and coagulation activation in sickle cell disease
Source: Res Pract Thromb Haemost. 2025 Sep 1;9(6):103168. doi: 10.1016/j.rpth.2025.103168 (PMC12538053; doi:10.1016/j.rpth.2025.103168)
Supplement: Supplementary Figures [file mmc1.pdf]

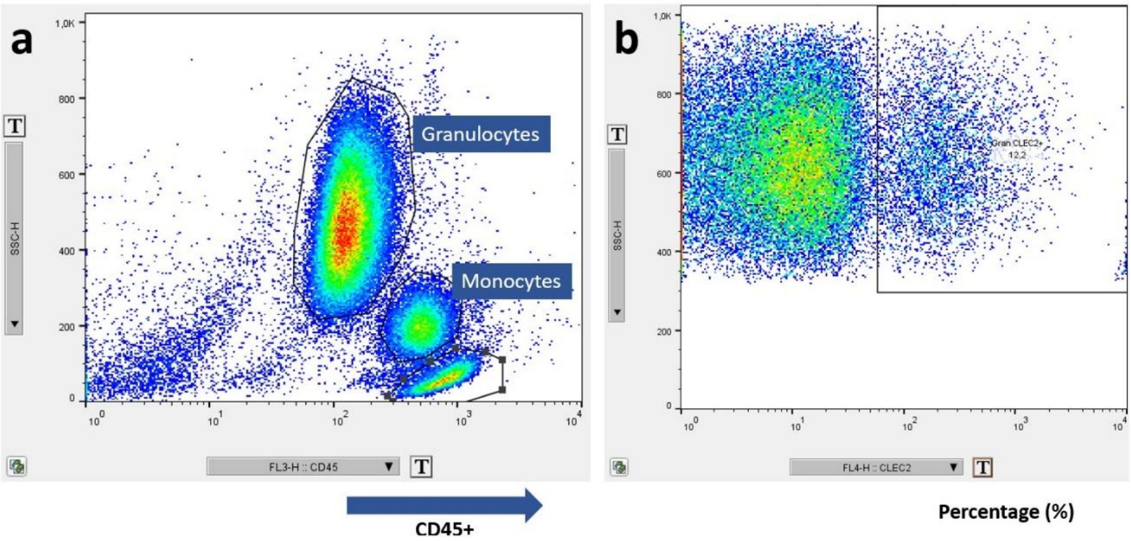

Supplementary figure 1: Separation of cell populations and assessment of expression by percentage in flow cytometry. (a) Granulocytic and monocytic cells were separated from CD45+ cells based on size and granularity. (b) From the CD45+ gates (granulocytes and monocytes), expression was evaluated by percentage.

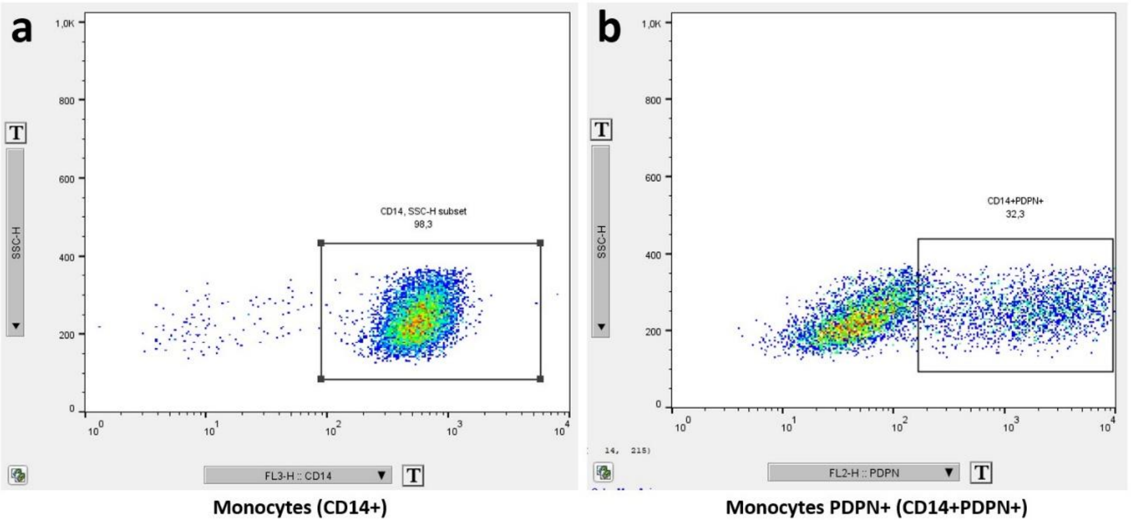

Supplementary figure 2: Evaluation of podoplanin (PDPN) expression in monocytes after three days of culture. (a) Monocytes were confirmed by CD14+ expression. (b) PDPN expression in monocytes (CD14+), with an average expression of 30%.
